# Cell Seeding Process Experiment and Simulation on Three-Dimensional Polyhedron and Cross-Link Design Scaffolds

**DOI:** 10.3389/fbioe.2020.00104

**Published:** 2020-03-04

**Authors:** Ziyu Liu, Maryam Tamaddon, Yingying Gu, Jianshu Yu, Nan Xu, Fangli Gang, Xiaodan Sun, Chaozong Liu

**Affiliations:** ^1^Division of Surgery and Interventional Science, Royal National Orthopaedic Hospital, University College London, London, United Kingdom; ^2^State Key Laboratory of New Ceramics and Fine Processing, School of Materials Science and Engineering, Tsinghua University, Beijing, China; ^3^Key Laboratory of Advanced Materials of Ministry of Education of China, School of Materials Science and Engineering, Tsinghua University, Beijing, China

**Keywords:** cell seeding, scaffold, cell distribution, simulation, DPM model

## Abstract

Cell attachment to a scaffold is a significant step toward successful tissue engineering. Cell seeding is the first stage of cell attachment, and its efficiency and distribution can affect the final biological performance of the scaffold. One of the contributing factors to maximize cell seeding efficiency and consequently cell attachment is the design of the scaffold. In this study, we investigated the optimum scaffold structure using two designs – truncated octahedron (TO) structure and cubic structure – for cell attachment. A simulation approach, by ANSYS Fluent coupling the volume of fluid (VOF) model, discrete phase model (DPM), and cell impingement model (CIM), was developed for cell seeding process in scaffold, and the results were validated with *in vitro* cell culture assays. Our observations suggest that both designs showed a gradual lateral variation of attached cells, and live cell movements are extremely slow by diffusion only while dead cells cannot move without external force. The simulation approaches supply a more accurate model to simulate cell adhesion for three-dimensional structures. As the initial stages of cell attachment *in vivo* are hard to observe, this novel method provides an opportunity to predict cell distribution, thereby helping to optimize scaffold structures. As tissue formation is highly related to cell distribution, this model may help researchers predict the effect of applied scaffold and reduce the number of animal testing.

## Introduction

In regenerative approaches, final tissue formation is strongly related to initial cell attachment (Wendt et al., 2006; Santoro et al., 2010). Preceding all other steps of tissue engineering, a better cell adhesion and an even spatial distribution are associated with improved culture results (Olivares and Lacroix, 2012). Although this is one of the determinants of the final bio-performance of a scaffold, it is difficult to observe the cell seeding process and cell distribution within the scaffold during the seeding process.

The lack of information about the cell attachment is especially pronounced for larger 3D structures such as scaffolds, since examination facilities for cell adhesion and spatial distribution are generally only available for 2D surfaces or thinner 3D structures.

Cell attachment is influenced by several scaffold properties including material, mechanical properties, and geometry. For example, geometry (porosity, pore size, tortuosity, and connectivity) can affect nutrient transport and cell ingrowth (Bancroft et al., 2002; Mastrogiacomo et al., 2006). Different manufacturing processes offer a wide range of possibilities to control these parameters. However, there are some contradictory parameters in scaffold design including the pore size with surface area, porosity with strength, and fatigue life, which lead to a complex assessment of the scaffold design and an increase in the number of *in vivo* tests for design optimization.

As *in vitro*, *in vivo*, and clinical tests usually require a large investment, numerical models offer a more ethical and economical alternative and controllable choice to investigate cell attachment to scaffolds. However, accuracy of prediction of cell seeding still needs to be improved. Byrne et al. (2007) modeled cell proliferation, migration, and differentiation by assuming that initially 1% of cells were randomly seeded in each lattice of the scaffold. However, the fact is that the cells’ positions at the initial stage are not random because the position of blood vessels and the physical impact all influence cell distribution. If we consider osteochondral (cartilage and underlying bone) tissue engineering as an example, the diseased tissue is removed and a scaffold is inserted to fill the void. To accurately predict the initial cell–scaffold interactions, we have to consider that, for example, during the operation, bone marrow-containing cells may flow into the osteochondral defect from the subchondral bone and that any physical movement of the scaffold influences cell distribution. In this case, finding the cell position at the initial stage in a precise model to simulate cell distribution and density on a three-dimensional scaffold at the beginning of an *in vivo* test is crucial.

Numerical methods for prediction of cell seeding efficiency should consider cell adhesion and fluid properties (speed and viscosity) (Wendt et al., 2003, 2009; Alvarez-Barreto et al., 2007; Koch et al., 2010). Xu et al. (2008) concluded that fluid velocities and shear stresses influence cell seeding density on a scaffold with random architecture. However, only considering culture medium’s velocity and shear stress to investigate cell attachment process is not enough. Each cell has its individual movement, which cannot be neglected and is as important as the fluid flow.

To improve the accuracy of prediction of cell seeding, an understanding of the type of cell–material interaction is crucial. Euler–Lagrange numerical approach, using Eulerian method to describe mediums and Lagrangian method to describe cell movement, would be a good solution. Olivares and Lacroix used Eulerian wall film model followed by Euler–Lagrange approach to simulate cell seeding process assuming that only one situation occurs when a cell impinges a wall and each cell is seen as spherical and trapped by the scaffold after it touches the material surface (Olivares and Lacroix, 2012).

In reality, cells can bounce back from the wall or sometimes split to several smaller particles when they approach the wall. The impinge types are similar to spray droplets interaction with engine combustion internal walls. The Stanton–Rutland model is mainly used to simulate internal combustion engines, but it also has potential to simulate the cell seeding process, in which cells can be considered as particles (O’Rourke and Amsden, 1996; Stanton and Rutland, 1996). The great advantage of this model is that when cells impinge a scaffold wall, four regimes (stick, rebound, spread, and splash) are considered (O’Rourke and Amsden, 2000). This novel method can interact with both the discrete phase model (DPM) and the Eulerian multiphase model. In the Eulerian multiphase interaction, the first phase represents the fluid (medium solution) and the secondary phase represents cells that can be captured by wall surfaces.

This paper provides a novel methodology with high accuracy to predict cell distribution and density on 3D-printed titanium alloy scaffold widely used for bone regeneration (Albrektsson et al., 1983; Hayashi et al., 1991; Urban et al., 1996).

To understand the details of the cell seeding process and the effects of scaffold design, two structures were investigated by both experimental and simulation approached, including a traditional scaffold structure called cubic (Heinl et al., 2008; Parthasarathy et al., 2010; Sallica-Leva et al., 2013; Li et al., 2014; Ahmadi et al., 2015) and the other a truncated octahedron (TO) (Chantarapanich et al., 2012; Amin Yavari et al., 2015; Hedayati et al., 2017). To exclude the effects of biochemical and biological parameters, the same material and manufacturing techniques were used for both designs. For simulating the cell distribution in 3D, the volume of fluid (VOF) model, designed for immiscible fluids having clear interface, is used to simulate solution filling in the scaffold, while DPM, which follows the Eulerian–Lagrangian approach, is used to trace cell movement during the cell seeding process. This approach includes the cell–material interaction scenarios including stick, rebound, spread, and splash and can predict the initial stage of cell attachment of *in vivo* or clinical test more accurately, leading to potentially a smaller number of experiments.

## Materials and Methods

### Scaffold Design and Manufacture

Titanium powder (EOSINT), created especially for EOSINT M addictive manufacturing system with a grain size distribution of 20–63 μm and density of 4.41 g/cm^3^, was used. The chemical composition of the powder is shown in Table 1.

**TABLE 1:** The chemical composition of EOS Titanium Ti64 powder (EOS art. no. 9011-0014).

%	Al	V	O	N	C	H	Fe	Ti
Normal value	5–6.75	3.5–4.5	<0.2	<0.05	<0.08	<0.015	<0.3	Balance

**FIGURE 1:**
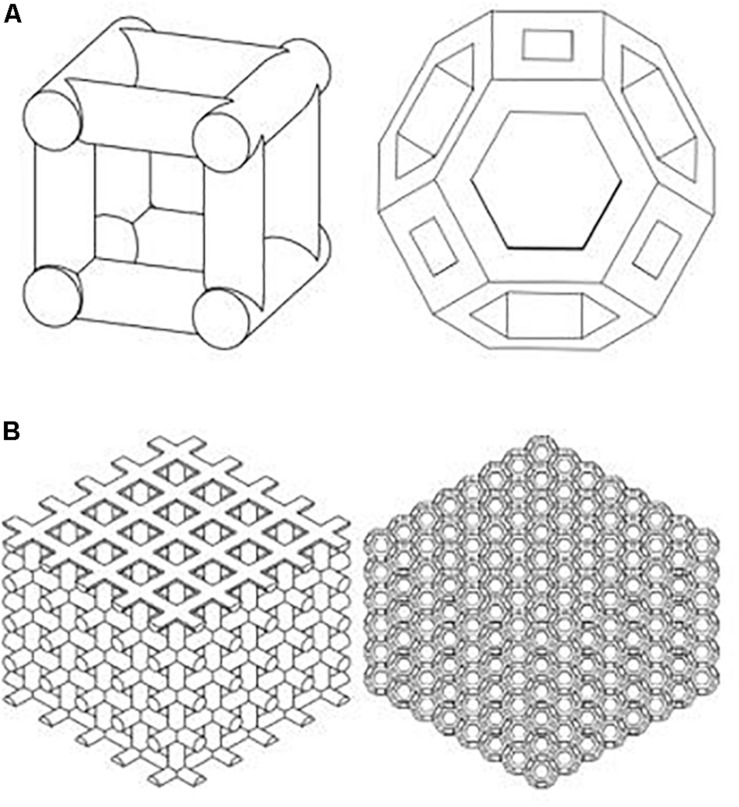
Cubic and truncated octahedron structures were used for titanium scaffold structure [**(A)** Unit cell; **(B)** scaffold].

For comparison, two types of scaffolds are manufactured by EOS M290 machine using the Direct Metal Laser Sintering (DMLS) method. An Yb fiber laser (400 W) with a scanning speed up to 7.0 m/s was used. Variable focus diameter was 100 μm, while inert gas supply was operated at 7000 hPa with 20 m^3^/h. The samples were then treated with a stress relief treatment at 650°C normalizing and annealing after 3 h. Cubic scaffold is a 3D structure characterized by six square faces each having six faces and 12 edges, while the TO scaffold is characterized by 14 faces (eight hexagonal and six square faces) and 36 edges. The cubic scaffold geometry is a cross-link design connected by a 0.5-mm-diameter cylinder. TO design is made by extruding a cut of an octagonal prism (formed by square sides and two regular octagon caps). The distance from each hole side to the edges is 0.1 mm. Both structures are designed as 7.9 mm × 7.9 mm length and width at 6 mm height. Two porous structures of cubic and TO unit cells are shown in Figure 1.

Porosity is determined by the apparent scaffold volume and the volume of the scaffold materials, which is given below (Eq. 1):

(1) $$P\,o\,r\,o\,s\,i\,t\,y=\left(1-\frac{V_{s\,t\,r\,u\,c\,t\,u\,r\,e}}{V_{o\,v\,e\,r\,a\,l\,l}}\right)\times 100\%$$

Where *V*_*structure*_ and *V*_*overall*_ are structural material’s volume and total volume of the scaffold, respectively. Specific surface area (SSA, abbreviated SA:V), is defined as the total surface area per unit of mass (m^2^/kg or m^2^/g) or volume (m^2^/m^3^). It is a fundamental biological concept because many bio-functions happen on the surface. The ratio can examine physical structures’ biological qualities such as internal nutrient transportations. Mathematically, it can be written as (Eq. 2):

(2) $$S\,A:V=\frac{S}{V}$$

*S* is the surface area of the scaffold and *V* is the volume of the scaffold. The physical dimension is L^–1^ (inverse length). This parameter can be used to measure and optimize physical structure as cell growth and mass transports are based on large surface areas.

### *In vitro* Cell Seeding Experiments

#### Cell Culture

All cells and culture reagents were purchased from Cyagen Biosciences Inc., China unless otherwise stated. The Sprague-Dawley (SD) rat mesenchymal stem cells (MSCs) were cultured in a growth medium (10% fetal bovine serum, 1% penicillin–streptomycin, and 1% glutamine). Medium was changed every 48 h. The cells were cultured in a humidified incubator with 5% CO_2_ at 37°C until they reached 80–90% confluency. Passage 6 was used for all experiments.

#### Cell Seeding

Before the cell seeding process, the scaffolds (*n* = 3 for each type) were placed in 48-well plates (Corning, United States) with 70% ethanol and were illuminated by UV light for 48 h. After disinfection treatment, scaffolds were rinsed by PBS and then put in the basal medium for 6 h. Next, 200,000 cells were suspended in 0.5 ml of medium and were gradually injected manually from the middle of the well wall in 3 s. After 6–12 h, the attachment was assessed by live/dead cell viability assay.

#### Cell Attachment/Viability Evaluation

A live/dead staining kit, containing Calcein-AM and propidium iodide (PI) solutions, was used for fluorescence staining of live and dead cells. The cell culture medium in each well was aspirated and the scaffolds were incubated with 2 μmol/L of Calcein-AM and 4 μmol/L of PI in phosphate buffer saline (PBS) at a 37°C incubator for 15 min and then observed under a Zeiss LSM710 Meta Confocal Microscope. The projecting data of cell distribution by projection imaging principles were analyzed by MATLAB R2017a program.

### Simulation of Cell Seeding Process

#### Simulation Framework

**FIGURE 2:**
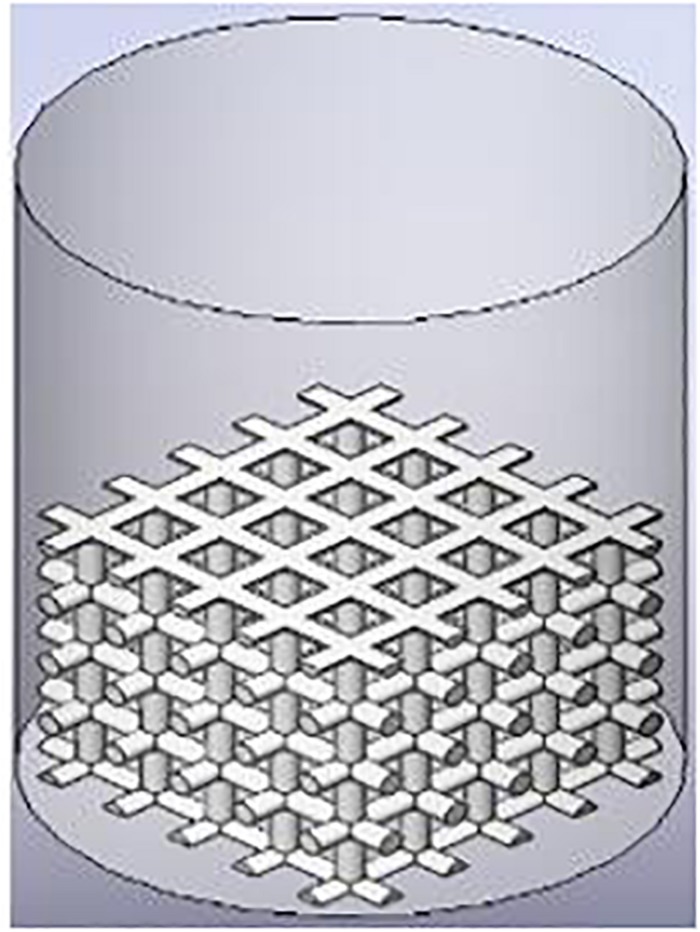
Illustration of test and simulation framework.

The culture medium is considered as continuity fluid while cells are considered as discrete particles. The VOF model, designed for immiscible fluids having clear interface, is used to simulate the culture medium filling process from the injection point to the container (well plate), and DPM by the Eulerian–Lagrangian approach is used to simulate cell movement during the cell seeding process. The cell impingement model (CIM), using the Stanton and Rutland impinge model, simulates the cell–wall interaction, which is governed not only by cell physical properties (viscosity, surface tension, and density) but also by the impingement conditions (cell velocity and diameter), and is shown in Figure 2.

In the VOF model, as the fluid phase is incompressible, the continuity equation can be simplified to a volume continuity equation as continuity equation (Eq. 3) with Navier–Stokes equation (Eq. 4). Two phases were set in the multiphase model, air and solution, as these two immiscible fluids can be separated clearly by interface. Gravitational forces can be neglected, and the second phase occupies low volume fraction.

(3) $$\nabla \cdot \vec{\text{u}}=0$$

(4) $$\rho \,\left(\frac{\partial \text{u}}{\partial \text{t}}+u\cdot \nabla \text{u}\right)=-\nabla \text{p}+\text{p}\,\vec{\text{g}}+\mu \,\nabla^{2}\text{u}$$

Where ∇ is divergence that represents quantity of the vector field source for each position, **u** is the flow velocity, ρ is the density of the fluid, **p** is the pressure, and **t** is the time.

In the DPM model, discrete particles representing the cells were carried by the fluid phase and trajectories of cells were predicted by integrating the force balance on cell written in a Lagrangian reference frame. All particles are set as non-rotating. Particle impingement causes energy loss because of the inelastic collision. The cell inertia with the force on the cell equates the force balance, and thus the equation can be expressed as (Eq. 5):

(5) $$\frac{\text{d}\,\vec{{\text{u}}_{\mathrm{cell}}}}{\text{dt}}=\frac{\vec{{\text{u}}_{\mathrm{fluid}}}-\vec{{\text{u}}_{\mathrm{cell}}}}{\tau_{r}}+\frac{\vec{\text{g}}\,\left(\rho_{\mathrm{cell}}-\rho_{\mathrm{fluid}}\right)}{\rho_{\mathrm{cell}}}+\vec{\text{F}}$$

Where $\vec{\text{F}}$ is defined as the force/unit particle mass as an additional acceleration and $\frac{\vec{u_{\mathrm{fluid}}}-\vec{u_{\mathrm{cell}}}}{\tau_{r}}$ is defined as the drag force per unit particle mass. *ρ*_****fluid****_ is the fluid density and *ρ*_****cell****_ is the cell density. $\vec{{\text{u}}_{\mathrm{cell}}}$ is the velocity of cells and $\vec{{\text{u}}_{\mathrm{fluid}}}$ is the fluid phase velocity. *τ*_****r****_, the particle relaxation time, as the main factor to govern cell motion, is expressed as Eq. 6 (Gosman and Ioannides, 1983).

(6) $$\tau_{r}=\frac{4\,\rho_{\mathrm{cell}}\,{\text{d}}_{\mathrm{cell}}^{2}}{3\,\mu \,{\text{C}}_{D}\,\text{Re}}$$

Here, μ is the molecular viscosity of the fluid, **d_cell_** is the particle diameter, **Re** is the Reynolds number, shown in Eq. 7, and **C_D_** is the drag coefficient and the equation is shown in Eq. 8.

(7) $$\text{Re}=\frac{\rho \,{\text{V}}_{\mathrm{pn}}\,{\text{d}}_{p}}{\mu }$$

(8) $${\text{C}}_{D}={\text{a}}_{1}+\frac{{\text{a}}_{2}}{\text{Re}}+\frac{{\text{a}}_{3}}{{\text{Re}}^{2}}$$

The constants *a*_1_, *a*_2_, and *a*_3_ are determined by the Reynolds number [27].

**FIGURE 3:**
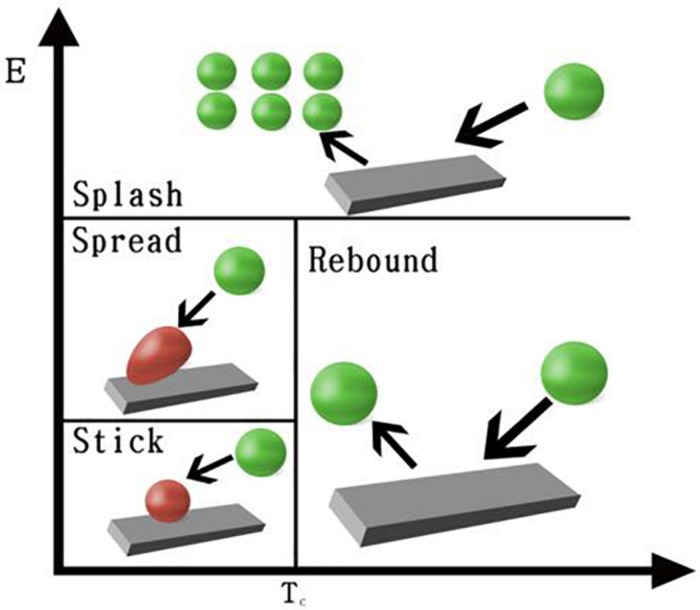
CIM model and impinge regimes definition.

In the CIM model, the cell seeding process by four regimes, including stick, rebound, spread, and splash, is considered to describe cell–material interaction when cells impinge a scaffold wall. In the stick regime, the cells that impact the scaffold can remain in their original spherical shape. In the rebound regime, cells leave the wall intact but with different velocities. In the spread regime, cells hit the wall and spread out to form a wall film. In the splash regime, parts of the particle attach to the wall and other parts leave the wall, as demonstrated in Figure 3.

Impinge regimes are defined by impact energy and critical transition temperature of the fluid, which can be written as:

(9) $${\text{E}}^{2}=\frac{\rho \,{\text{V}}_{\mathrm{pn}}^{2}\,{\text{d}}_{p}}{\sigma }\,\left(\frac{1}{\min\left(\frac{h_{o}}{d_{n}},1\right)+\frac{\delta_{\mathrm{bl}}}{d_{n}}}\right)$$

Where **E** represents impact energy (dimensionless), ρ is the seeding solution’s density, **V_pn_** is cell velocity (to the wall), **d_p_** is the diameter of each cell, σ is the surface tension of the solution, **h_o_** is the film height, and *δ*_****bl****_ is the boundary layer thickness.

Boundary layer thickness is calculated by:

(10) $$\delta_{\mathrm{bl}}=\frac{{\text{d}}_{p}}{\sqrt{\text{Re}}}$$

Critical transition temperature is written as:

(11) $${\text{T}}_{c}={\text{T}}_{c}^{*}\cdot {\text{T}}_{s}$$

Where **T_s_** is the saturate temperature and ${\text{T}}_{c}^{*}$ is the critical temperature factor, whose value is usually between 1 and 1.5. The value of ${\text{T}}_{c}^{*}$ is set as 1, and in that case, critical transition temperature is equal to the saturate temperature.

#### Cell Seeding Process Modeling and Boundary Conditions

The cell seeding process was simulated by ANSYS Fluent coupling the VOF model, DPM model, and CIM model.

In VOF simulation, two phases are computed individually during the fluid phase solving process. The main phase is cell solution and the other one is air. The solution’s surface tension coefficient is set as 0.07 n/m. k-epsilon Low-Re model is used and no turbulent viscosity is set. The nutrient solution is treated as a laminar and incompressible continuous fluid (with 1.0 × 10^3^ kg/m^3^ for the density and 0.001 Pa⋅s for the viscosity) that is calculated by the continuity equation and Navier–Stokes equation, while the dispersed phase is calculated by tracking particles (bone marrow MSCs) through the flow field.

In the DPM model, particle dispersion is calculated by tracking the particles (bone marrow MSCs) through the flow field. The injection speed of mass flow rate is 8.18 × 10^–10^ kg/s and the particles are injected to the system for 5 s from start time. Particles were seen as spherical (drag law is spherical) and diameter (with 1.0 × 10^3^kg/m^3^ for the density, 0.005 Pa⋅s for the viscosity, and 0.03 N/s for the surface tension) is set at 15 μm, which is the size assumption of SD-rat MSCs. The discrete random walk model is also used to govern particle movement. The well size is 15 mm in depth and 11 mm in diameter. Particles do not escape and well walls do not trap the cells. When cells impinge the wall, they were reflected and rebound back. Only the scaffold traps the cells. As for the impinge type control, we assumed a splash regime such that each cell could separate into six small liquid drops when they impinge the wall. Non-slip and non-adherence conditions are applied for the wall and scaffold.

In the CIM model, only physical impingements were involved in the cell seeding process. The stick regime occurs when **E** is less than 16, and the particle and the wall (scaffold surface) have the same velocity. The spreading regime occurs when **E** is between 57.7 and 16. The rebound regime occurs when the impact energy is below 57.7 and the wall temperature is above the critical transition temperature. In the splash regime, when the impact energy is above 57.7, the cell (particle) could be separated into several smaller particles (O’Rourke and Amsden, 1996; Stanton and Rutland, 1996). In this simulation, we assumed that it could be separated into six drops and the number should be further investigated by the experiments.

### Statistical Analysis

All statistical analysis was performed using Origin 2019 software. All quantitative data comparison is analyzed by statistical hypothesis testing and 0.05 was set as the *p* value.

### Cell Distributions Within the Scaffold in *in vitro* Cell Seeding

Pore size plays a very important role in the initial cell distribution, migration, and nutrient transport. Even the rate of cell growth and migration are determined by it (J.A. Sanz-Herrera et al., 2009). Some researchers suggest that 100–400-μm pore sizes are optimal for bone tissue regeneration (Hulbert et al., 1970; Schliephake et al., 1991; Bloebaum et al., 1994). Accordingly, two types of scaffolds, including TO structure and cubic structure, were designed, and the structure parameters are listed in Table 2.

**TABLE 2:** Geometry parameters of scaffolds.

	V (mm^3^)	S (mm^2^)	Porosity	Maximum pore length of side (μm)	Maximum pore diagonal length (μm)	Specific surface area
Truncated octahedron	60.54	1541.67	83.81%	240	480	25.47
Cubic design	72.65	598.40	80.57%	1000	1414	8.24

With the same macroscale parameter (length, width, and height) and nearly the same material volume and porosity, the TO scaffold was predicted to have a better bio-performance than a cubic design due to a larger surface area. Moreover, although TO has a bit less material volume and larger porosity, its SSA is three times as much as the cubic one, which means that TO provides more area for cell movement and adhesion and more space for nutrient transport.

**FIGURE 4:**
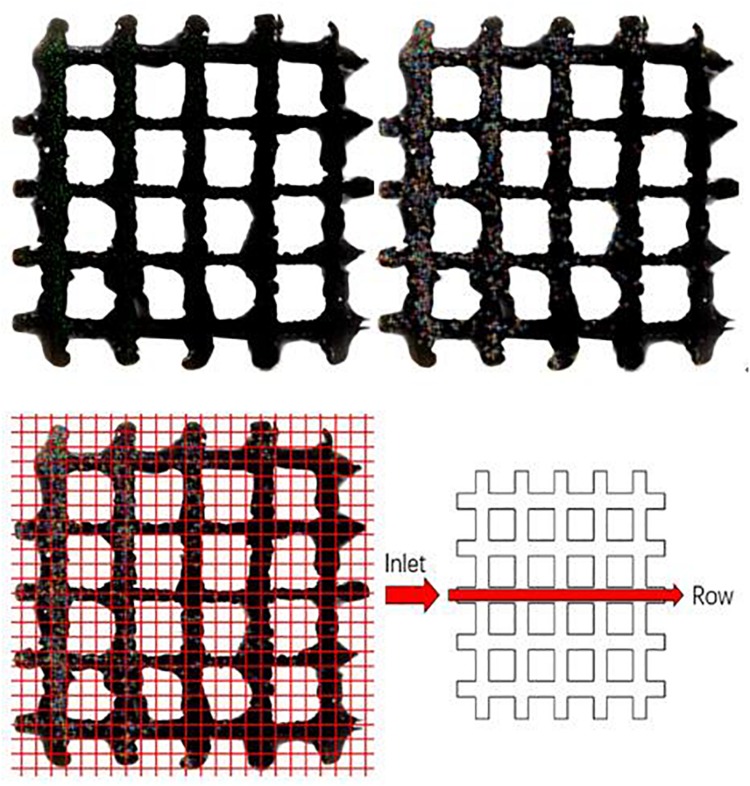
Cells growth on porous titanium matrix was examined by confocal microscopy, and the confocal image was further processed to determine the cells distribution.

Cell localization was analyzed by MATLAB R2017a program based on confocal microscope images (Figure 4A). The Calcein-AM and PI solutions were used for staining viable and dead cells, respectively. To separate viable (green) and dead (red) cells, the program first distinguishes the areas of each color and split it into two images. In each image, some of the cell adhesions and impurities are etched away as some cells overlap. Then, every detected cell is expanded and the resulting two images are combined into one image showing no difference between the before and after (Figure 4B). The cells are then localized (Figure 4C) by overlaying same-sized grids and the numbers are counted by row from the inlet position to the other side (Figure 4D).

**FIGURE 5:**
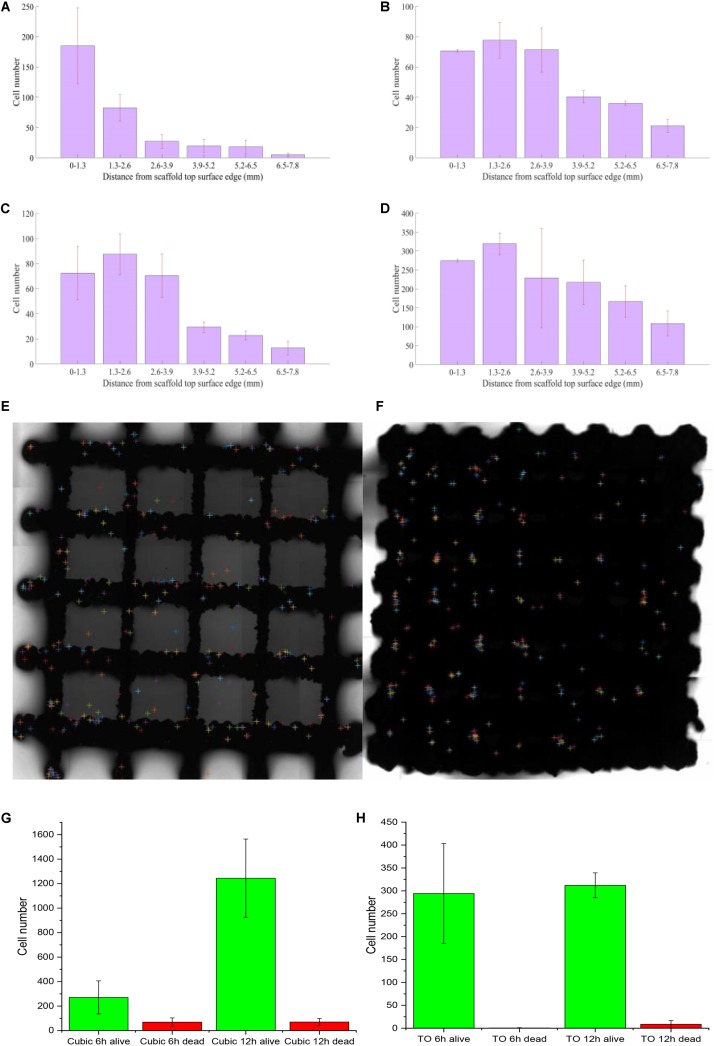
Attached cell numbers at the top surface of scaffolds [**(A)** 6 h on cubic scaffold; **(B)** 12 h on cubic scaffold; **(C)** 6 h on TO scaffold; **(D)** 12 h on TO scaffold; **(E)** cell distribution analysis at 6 h on cubic scaffold; **(F)** cell distribution analysis at 6 h on TO scaffold; **(G)** alive and dead cells on cubic scaffold all timeline; **(H)** alive and dead cells on TO scaffold all timeline]; *n* = 3 for each scaffold; error bars show standard deviation.

Looking at the cell distribution in both TO and cubic scaffolds, even with a highly interconnected pore structure and appropriate pore sizes, we observed that the cells cannot distribute evenly after 12 h. According to *t* test on cubic scaffold, the cell number around 0–1.3 mm showed significant difference compared to the other five ranges (*p* = 0.276; 0.112; 0.100; 0.099; 0.079 from the near side 1.3–2.6 to further 6.5–7.8 mm). The 1.3–2.6 and 2.6–3.9 mm ranges also showed significant difference (*p* = 0.137). Although three groups, in the range of 2.6–6.5 mm, did not show a significant difference in cell number, the fewest cell number group (5.2–6.5) showed a significant difference compared to the edge 6.5–7.8 mm (*p* = 0.415). As for the TO scaffold, there was no significant difference between these three ranges (0–3.9 mm) and also no significant difference between the 3.9–7.8 mm groups. However, the lowest group of 0–3.9 mm (2.6–3.9 mm) has significant difference with the highest group of 3.9–7.8 mm (3.9–5.2 mm), whose *p* value is 0.135. To sum up, both designs showed a gradual lateral variation of attached cells as shown in Figure 5. After the initial seeding by injection, it is also predicted that there is insufficient force to move the cells from the injection site elsewhere. This was confirmed by the results showing that most cells were inclined to attach on the injection side of the beams in both timelines. Across the surface of the scaffold, distribution of dead cells in the lateral direction follows the same trend, and there is nearly no difference between dead cell numbers after 6 and 12 h.

Cell concentration distribution was chosen to compare the cell seeding process in two types of scaffolds. From Figures 5A–D, we can see that there was a more even cell distribution in both structures from 6 h post-seeding to 12 h in all areas. After 12 h, both structures showed that there were more cells attached further away (1.3–2.6 mm) compared to the point of injection (0–1.3 mm). Even with a high number of interconnected pores, neither design could achieve a perfect cell distribution. We also observed nearly no difference between dead cell numbers at 6 and 12 h, showing a very high survival rate on both types of scaffold seen in Figures 5G,H.

However, the TO scaffold showed a more even distribution than the cubic design, which indicated that the TO scaffold structure is more suited for fluid flow and cell movement seen in Figures 5E,F. Cell numbers in the cubic scaffold at the 3.9–7.8-mm position showed a rapid increase from 6 to 12 h. Cell numbers in the TO scaffold at the 6.5–7.8-mm position showed double cell concentration. The results indicated that the cubic scaffold structure is suited for cell attachment.

### Simulation of Cell Seeding Process

**FIGURE 6:**
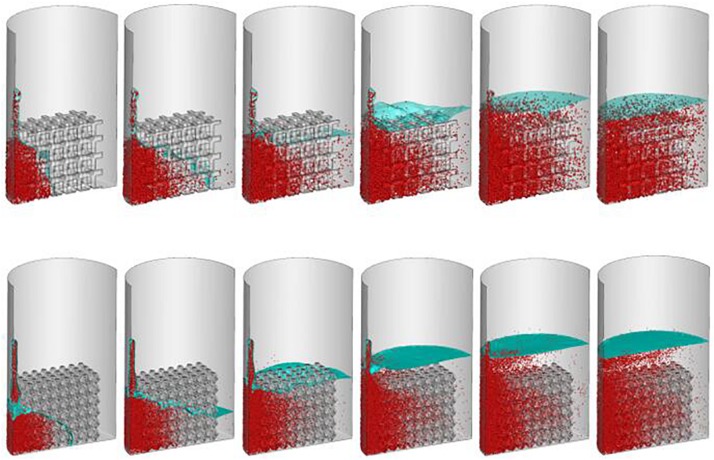
Predicted cell distribution during cell seeding process. From left to right represent the cells distribution at second one, two, three, four, five and six, respectively.

Simulating the cell seeding process is a computationally challenging problem requiring large grids to solve numbers of discrete particles. The solution and cell performance during the cell injecting process are shown at different time points (1–6 s) (Figure 6).

**FIGURE 7:**
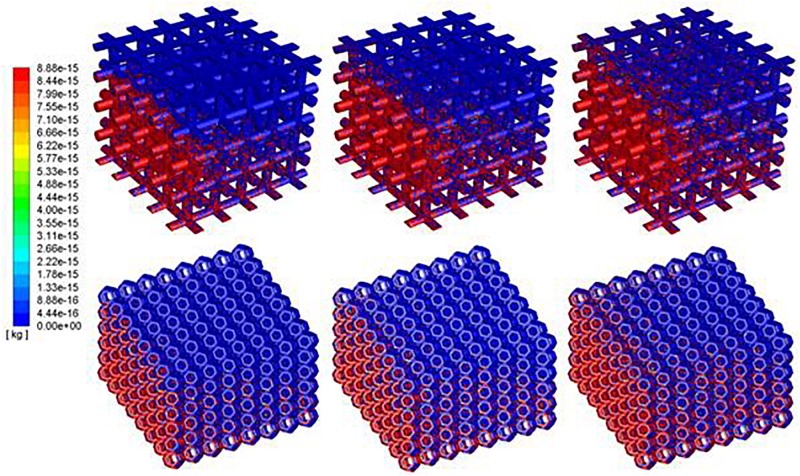
Predicted attached cell mass at second 3, 4 and 5 during cell seeding process. Top row: cubic design; Bottom row for truncated octahedron design.

The densities of cells attached to the TO scaffold are higher than those attached to the cubic structure (Figure 7). For both structures during the injection/seeding time, the numbers of cells increase rapidly, and the cells flow from the injection point to the surrounding areas.

To quantitate the cell density relationship between deposited cells by scaffold and injected cells, cell seeding efficiency (Φ) is used to describe the percentage of the attached cells (Na) over the injected cells (Ni) (see Eq. 12).

(12) $$\Phi =\frac{{\text{N}}_{a}}{{\text{N}}_{i}}\times 100\%$$

**FIGURE 8:**
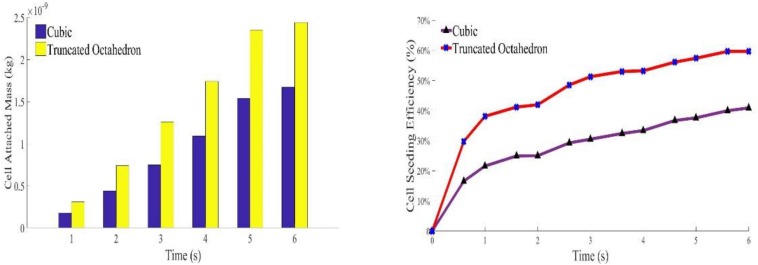
Variation of calculated attached cell mass during sell seeding process.

With the same material volume, it was shown that cell density in TO always has a higher rate than the cubic scaffold. It is because the TO structure has more surface area than the cubic one, which could provide more opportunities for cell to attach. As the injection speed of solution is slow and gradual, no splash regime occurs during the whole process, which means no cell death because of the splash regime. As there is no evidence and experiments have been done to explore whether the rebound regime will cause cell death or not, it is assumed in this program that rebound would not cause cell death. As for cell seeding efficiency, cells were impinging the walls and attached to the scaffold very quickly in the first few seconds. The fluid, flowing into the system around this time, does not carry enough cells to the opposite side of the scaffold. The flowing fluid though has enough cells, as the beams at the injection side were nearly saturated, and most of these cells were attached to the beams that were close to the injection side. In that case, the attachment speed would decrease as the fluid needs time to flow across the saturated beams. That is why the cell seeding efficiency of both structures reaches a plateau around 2 s (Figure 8). After that, the cell seeding efficiency rises gradually and becomes steady after 5.8 s. The simulation results coincide with the test results that show that cell distribution in the TO scaffold is more uniform than that in the cubic scaffold.

**FIGURE 9:**
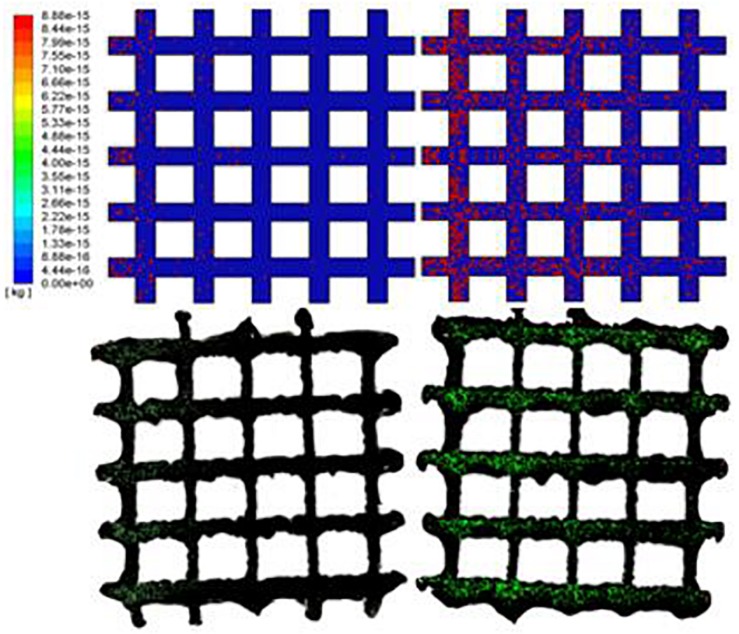
The predicted cell distributions in the scaffold are in line with the experimental results as confirmed by confocal examinations (6 and 12 hours time points).

To better validate the cell distribution longitudinally and transversely, the two different time points (4.2 and 5.4 s for simulation; 6 and 12 h for the experiment) were selected, given in Figure 9, and are discussed in detail in see Section “Conclusion.”

## Discussion

A scaffold with a porous structure increases tissue ingrowth, nutrient transport, and integration with the host tissue (Wu et al., 2014). To facilitate the desired tissue regeneration, the structural design of the scaffold often considers porosity, pore size and shape, orientation of the interconnected channel, and hierarchical control of the structure. The TO scaffold, with nearly the same material volume as the cubic design scaffold with three times larger specific area, is predicted to have a better cell attachment performance. The experimental results indicated that the TO scaffold showed a more even distribution than the cubic design after 6 h.

The novel model showed the ability of spatial cell distribution prediction through multiphase approach by treating cells as particles. The simulation results were qualitatively and quantitatively comparable with the experimental results under the same conditions. Both experimental and simulation results showed that cell distribution has a gradual decrease from the injection site and most of the cells preferred to attach on the injection side areas as there is insufficient force to move the cells from the injection side to the opposite side.

The temporal changes in cell distribution were minor, which hints that the cell movements are extremely slow by diffusion only. Experimentally, there were no differences in dead cells at 6 and 12 h. The reason could be that most cell deaths were caused by cell impingement at the initial stage of seeding and they could not be moved without an external force. Smaller beams in TO scaffold would supply enough drag for solution movement and cell speed would also decrease, which would cause fewer dead cells on the cubic structure than the TO scaffold.

Spatially distributed beams could provide more drags on the fluid, decreasing its velocity, which may reduce the chance of cell death. Both the TO and cubic scaffold are designed with the same length, width, and height. The prediction showed that the TO scaffold, with lower material volume but more surface area, should have a greater attached number of cells. This was confirmed as the cell seeding efficiency in the TO was nearly 60%, whereas it was just slightly more than 40% in the cubic scaffold.

Dunn et al. discovered that although the initial cells on the scaffold were evenly distributed and the cell density was increased in all areas after 10 days of culture, there was an obvious decrease in cell density from the outside toward the center of the scaffold (Dunn et al., 2006). High rates of nutrients were absorbed by the cells at the boundary and high cell density resisted nutrient transportation. In that case, the TO scaffold with spatial distribution beams could provide a more suitable environment for nutrient transport and cell distribution. Moreover, morphology of the pores is also related to cell distribution and fluid flow and it needs to be further investigated.

Compared to the model mainly used for cell distribution (Olivares and Lacroix, 2012), which treats every impinged cell to the solid surface as “absorbed/trapped,” our advanced model includes more impinge types like rebound and splash. Moreover, the trap model does not consider the idea that the liquid film can be stripped by the movement of the fluid flow, which always happens during the process. Another theory to simulate the cell seeding process is that the cellular system would achieve the minimum energy state through the Metropolis Monte Carlo method (Robu et al., 2011). This method showed that majority of the cells penetrate the scaffold without attachment and their model did not consider the influence of scaffold material, solution density, surface tension, etc.

Scaffold implant is a promising alternative treatment for osteoarthritis patients (V. Karageorgiou and Kaplan, 2005). Cell adhesion to implant material could be one of the key steps for bone and cartilage regeneration (Wendt et al., 2006; Santoro et al., 2010). To reduce the number of *in vivo* and animal tests, this method, combined with cell proliferation, differentiation, and migration model (Søballe et al., 1992; Huiskes et al., 1997; Byrne et al., 2007) in the future, would be able to predict cartilage and bone regeneration and their mechanical properties.

Furthermore, this model can also predict nutrient transport like oxygen and glucose. Although the density, viscosity, surface tension, and particle size will be different, the distribution of the particle will not change.

The main limitation of this new numerical method for tissue engineering is that the program only considers the cell as liquid droplet. If cell seeding speed is very slow, the splash regime would not occur. If one cell splashes into several particles after impinging the scaffold, all of them are treated as dead cells. In reality, only one particle should be seen as cell staying at the scaffold and other particles should be seen as droplet or solution. In other words, this program is suitable for low-speed cell attachment prediction and further experiments should be done to explore the suitable equation for governing splash regime. Furthermore, during the interaction with the Eulerian wall film model, additional particles may be created when the shear stress is large enough to be stripped from the film. These discrete particles could cause splash or rebound circumstances and may be absorbed by the wall afterward. However, cells are not guaranteed to be alive after these movements. Moreover, in this study, we only considered one of the main influencing factors for cell seeding, which is scaffold geometry. To make this model more accurate for predicting cell attachment in more conditions, the fabrication of the scaffold and the material surface morphology and biomechanical feature should also be considered. An additional code could be written based on *in vitro* tests through live and dead cell spatial distribution. In that case, through different specific codes, this model could predict *in vitro* and *in vivo* tests for all materials.

## Conclusion

A simulation approach, by ANSYS Fluent coupling the VOF model, DPM model, and CIM model, was developed for cell seeding process in porous scaffolds. This novel methodology can predict the cell distribution in scaffold and assess the design of a scaffold. The TO design showed a more even cell distribution than the cubic design by providing a more suitable environment for nutrient transport and cell movement and distribution. Live cell movement is extremely slow by diffusion only while dead cells cannot move without external force.

## Data Availability Statement

The datasets analyzed in this article are not publicly available. Requests to access the datasets should be directed to CL, chaozong.liu@ucl.ac.uk.

## Author Contributions

ZL, MT, and JY did the proofreading of this manuscript. YG, NX, and FG helped to do the cell seeding work and cell staining. XS provided all the facilities for the cell work. CL provided all the funding for this manuscript.

## Conflict of Interest

The authors declare that the research was conducted in the absence of any commercial or financial relationships that could be construed as a potential conflict of interest.
